# Non-ST Elevation Myocardial Infarction Following Significant Weight Loss During Tirzepatide Therapy: A Case Report

**DOI:** 10.7759/cureus.117278

**Published:** 2026-10-01

**Authors:** Aparajita Roy, Umesh Kumar Pabani, Argyris Kyriakou, Mark K Elliott

**Affiliations:** 1 Cardiology, King George Hospital, London, GBR

**Keywords:** acute coronary syndrome, coronary artery disease, glp-1 receptor agonist, mounjaro, non-st elevation myocardial infarction, tirzepatide, weight loss

## Abstract

Tirzepatide is a dual glucose-dependent insulinotropic polypeptide (GIP) and glucagon-like peptide-1 (GLP-1) receptor agonist that produces substantial weight loss and improves multiple cardiometabolic risk factors. Current evidence supports its cardiovascular safety, with no established association between licensed tirzepatide therapy and acute coronary syndrome (ACS). As its clinical use continues to expand, reporting unusual cardiovascular presentations remains important for pharmacovigilance. We present a case of a 48-year-old South Asian man with obesity, diet-controlled prediabetes, a history of smoking, and a family history of premature coronary artery disease who had been receiving tirzepatide for approximately 18 months, achieving a weight loss of approximately 30 kg. Following increased physical activity, he developed progressive exertional chest pain and dyspnoea. Electrocardiography demonstrated inferolateral ischaemic changes, and serial high-sensitivity troponin T measurements were consistent with a non-ST elevation myocardial infarction (NSTEMI). Transthoracic echocardiography showed preserved left ventricular systolic function with inferior regional wall motion abnormalities. Coronary angiography revealed severe triple-vessel coronary artery disease involving the left anterior descending, left circumflex, and right coronary arteries. He subsequently underwent successful coronary artery bypass grafting with an uncomplicated postoperative recovery. Although a temporal association existed between prolonged tirzepatide therapy, substantial weight loss, and presentation with NSTEMI, the presence of advanced multivessel coronary artery disease suggests longstanding atherosclerosis rather than a direct adverse effect of tirzepatide. A more plausible explanation is that significant weight loss improved functional capacity, allowing increased physical activity that unmasked previously silent obstructive coronary artery disease. This interpretation is consistent with current evidence demonstrating favourable cardiovascular effects of tirzepatide while acknowledging that causality cannot be established from a single case. This case highlights the importance of thoroughly evaluating new exertional chest pain in patients receiving tirzepatide despite marked improvements in weight and metabolic health. Clinicians should remain vigilant for underlying coronary artery disease in individuals with traditional cardiovascular risk factors. This report describes a temporal association rather than a causal relationship and contributes to the growing body of real-world experience with tirzepatide.

## Introduction

Tirzepatide is a novel dual glucose-dependent insulinotropic polypeptide (GIP) and glucagon-like peptide-1 (GLP-1) receptor agonist approved for the treatment of type 2 diabetes mellitus and obesity [1]. By targeting both incretin pathways, tirzepatide produces substantial and sustained weight loss while improving glycaemic control and a range of cardiometabolic risk factors, including blood pressure, insulin resistance, and lipid profile [1]. In the SURMOUNT-1 trial, participants with obesity achieved mean weight reductions of up to 20% of baseline body weight after 72 weeks of treatment, establishing tirzepatide as one of the most effective pharmacological therapies currently available for obesity management [1].

Obesity is a well-established risk factor for atherosclerotic cardiovascular disease, and intentional weight reduction is associated with improvements in cardiovascular risk [2]. Consequently, tirzepatide is generally expected to confer cardiovascular benefit rather than harm. Recent cardiovascular outcome data demonstrated that tirzepatide was non-inferior to dulaglutide with respect to major adverse cardiovascular events in patients with type 2 diabetes and established cardiovascular disease, supporting its cardiovascular safety profile [3].

Despite its favourable cardiometabolic effects, increasing use of tirzepatide in routine clinical practice has led to recognition of uncommon adverse events that were not fully characterised in pre-licensing trials. Although gastrointestinal adverse events remain the most commonly reported adverse effects of tirzepatide, experience with its long-term cardiovascular safety in routine clinical practice continues to evolve. Published reports describing acute coronary syndrome (ACS) occurring during licensed tirzepatide therapy remain extremely limited [4]. However, whether these represent causal drug effects, coincidental occurrences in high-risk individuals, or the clinical unmasking of previously silent coronary artery disease remains uncertain. At present, there is little published literature describing ACS occurring after substantial weight loss achieved with licensed tirzepatide therapy.

We present the case of a 48-year-old man who experienced significant weight loss during long-term tirzepatide therapy before presenting with a non-ST elevation myocardial infarction (NSTEMI). Coronary angiography demonstrated previously undiagnosed severe triple-vessel coronary artery disease requiring urgent coronary artery bypass grafting. This case highlights the importance of maintaining a high index of suspicion for obstructive coronary artery disease in patients presenting with new exertional chest pain despite marked improvements in body weight and metabolic health during tirzepatide treatment. Rather than implying causality, this report describes a temporal association and discusses potential mechanisms, including the possibility that substantial weight loss and increased physical activity may unmask previously silent but clinically significant coronary artery disease.

## Case presentation

A 48-year-old South Asian man presented to the emergency department with a two-week history of exertional chest pain and progressive shortness of breath on exertion. His symptoms initially occurred during exercise at the gym, when he experienced sudden-onset central chest discomfort associated with dyspnoea that resolved with rest. Over the following week, he continued to experience intermittent exertional chest pain. He denied associated nausea, vomiting, palpitations, syncope, or symptoms suggestive of heart failure. As his symptoms persisted and he felt generally unwell over the preceding few days, he attended hospital for further assessment.

His medical history included obesity with significant weight loss following treatment with tirzepatide (Mounjaro), which he had been receiving privately for approximately 18 months. He had lost approximately 30 kg during treatment and was receiving a maintenance dose of 5 mg once weekly at presentation. His pre-treatment weight was 134 kg, and his BMI was 37.9 kg/m². He had diet-controlled prediabetes and was an ex-smoker with a 10-pack-year history (stopped two years before presentation) and had a significant family history of premature coronary artery disease, with his mother sustaining a myocardial infarction at 55 years of age. 

On examination, he was alert, orientated and haemodynamically stable. His temperature was 37.1°C, heart rate 68 beats/minute, blood pressure 120/67 mmHg, respiratory rate 19 breaths/minute and oxygen saturation 97% on room air. His weight was 104.3 kg and his height 188 cm, corresponding to a body mass index (BMI) of 29.5 kg/m². Cardiovascular and respiratory examinations were unremarkable, with no clinical evidence of heart failure.

The initial electrocardiogram (ECG) demonstrated sinus rhythm with inferolateral ischaemic changes, including T-wave inversion in the inferior and lateral leads as shown in Figure 1. These findings remained unchanged on a repeat ECG. High-sensitivity cardiac troponin T was elevated, with serial values of 288 ng/L, 369 ng/L, and a peak of 541 ng/L, consistent with a diagnosis of NSTEMI, as shown in Table 1. D-dimer was negative (<0.19 mg/L FEU), and computed tomography angiography of the thoracic aorta excluded acute aortic dissection.

**Figure 1:**
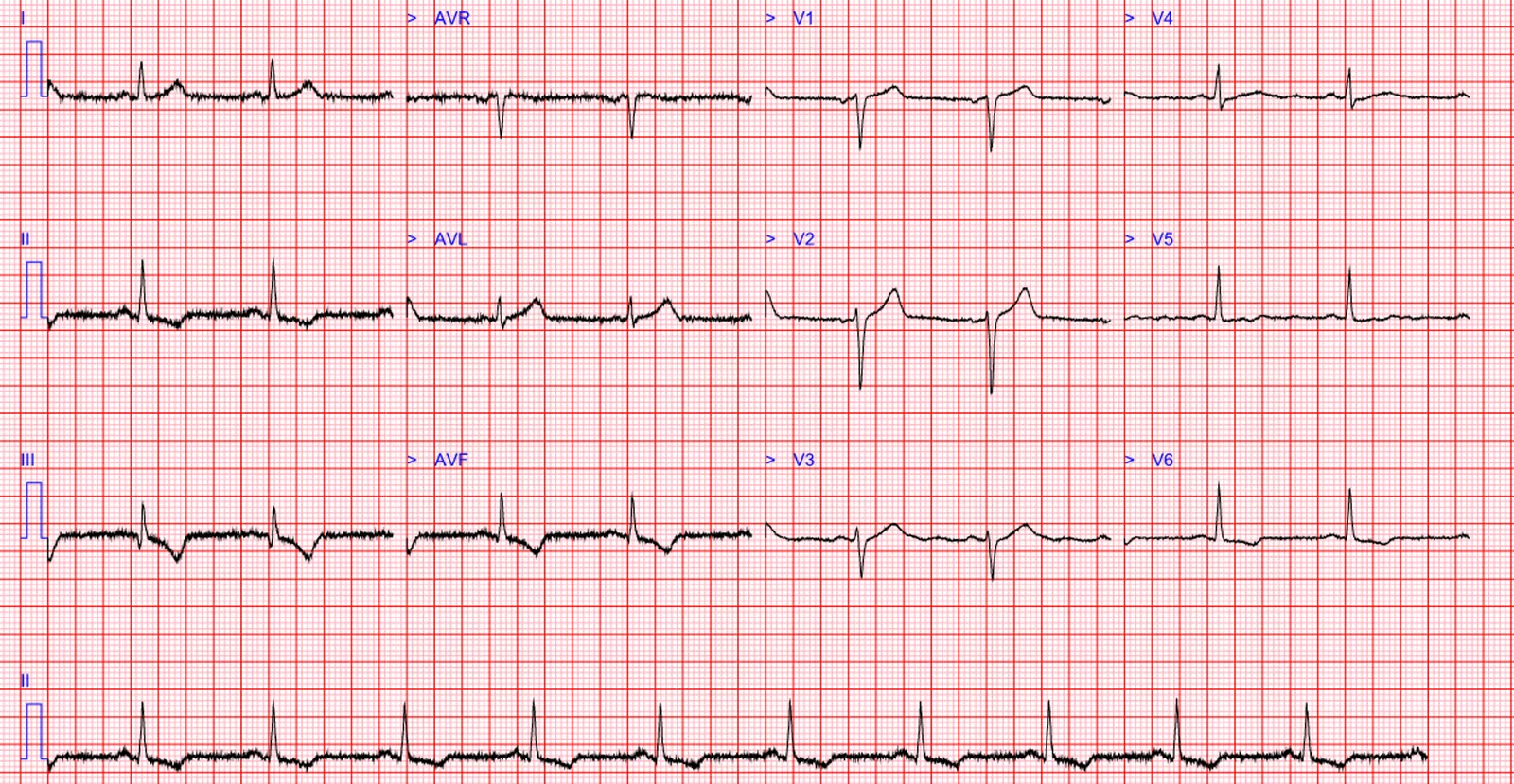
Admission 12-lead electrocardiogram demonstrating sinus rhythm with inferolateral ischaemic changes, including T-wave inversion in the inferior leads (II, III, aVF, V5 and V6) consistent with non-ST elevation myocardial infarction.

**Table 1:** The patient's blood reports at admission

Blood test	Results	Reference Range
Haemoglobin	165 g/L	133 -173 g/L
White cell count	5.9 × 109/L	3.8 -11 × 109/L
Haemoglobin A1C	31 mmol/mol	20 -41 mmol/mol
Total Cholesterol	2.4 mmol/L	0 -5 mmol/L
Low-Density Lipoprotein Cholesterol	1.1 mmol/L	
Cholesterol: High-Density Lipoprotein (HDL) Ratio	3.0	0-5
Triglycerides	1.09 mmol/L	0 -1.7 mmol/L
HDL Cholesterol	0.8 mmol/L	0.9-1.5 mmol/L
Creatinine	89 umol/L	59-104 umol/L
Troponin T (0 hour)	288 ng/L	<14 ng/L
Troponin T (3 hours)	369 ng/L	<14 ng/L
Troponin T (6 hours)	541 ng/L	<14 ng/L
D-dimers	<0.19 mg/L FEU	<0.19 mg/L FEU

Transthoracic echocardiography demonstrated a normal-sized left ventricle with preserved overall systolic function (estimated left ventricular ejection fraction approximately 55%). Regional wall motion abnormalities were present, with hypokinesia involving the basal-to-mid inferior and basal infero-septal segments. No significant valvular abnormalities were identified, and right ventricular size and function were normal. 

Coronary angiography demonstrated severe multivessel coronary artery disease. The left main stem was unobstructed. The left anterior descending artery contained a long segment of severe proximal-to-mid vessel disease (Figure 2). The dominant right coronary artery demonstrated severe disease within the mid and distal segments together with severe ostial disease affecting the posterolateral ventricular branch (Figure 3). The left circumflex artery showed mild irregularities within the atrioventricular circumflex vessel and moderate-to-severe disease involving the first obtuse marginal branch (Figure 4).

**Figure 2:**
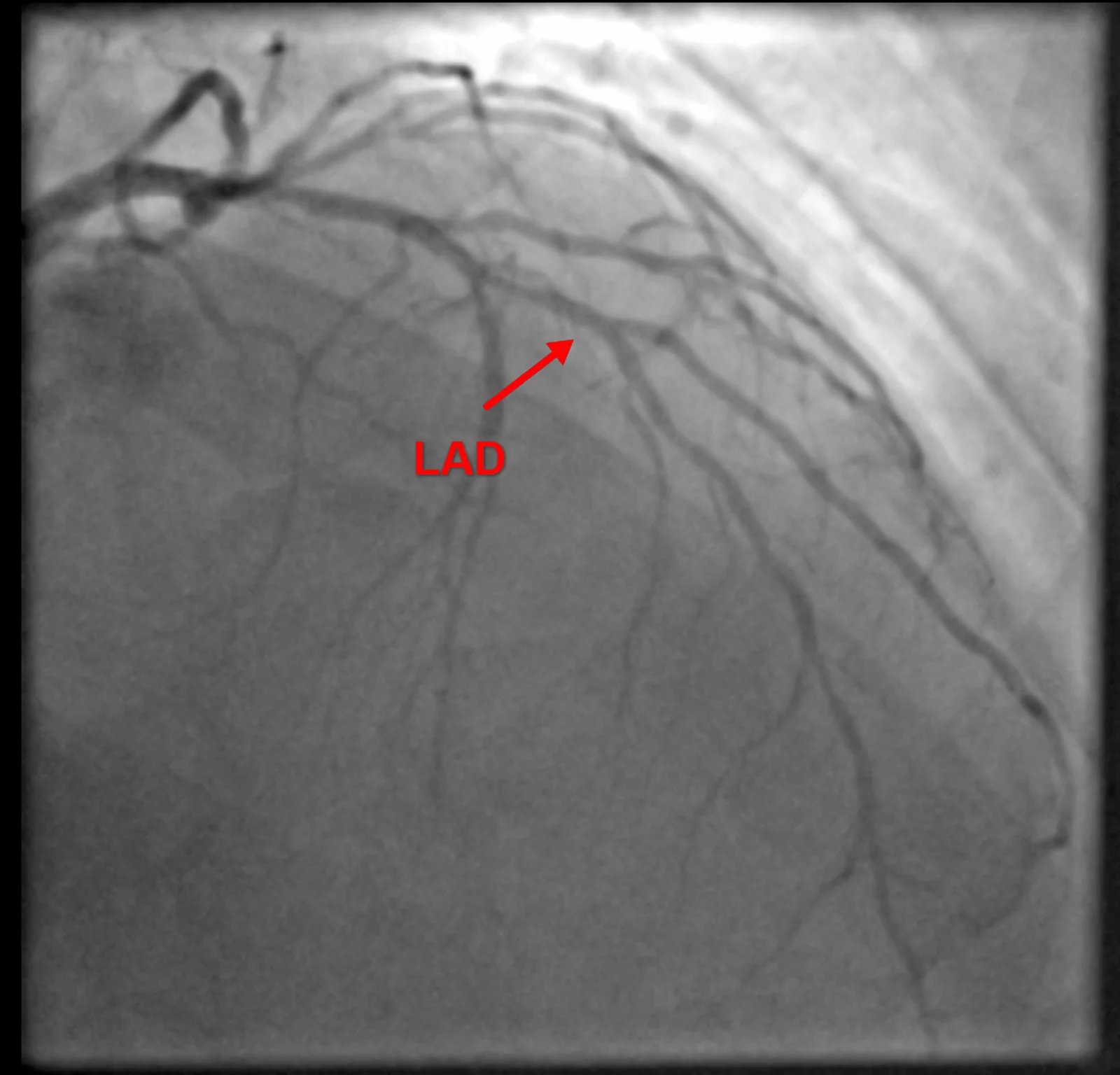
Diagnostic coronary angiography of the left coronary system demonstrating diffuse severe disease involving the left anterior descending artery (LAD) as pointed out by red arrow. The left main stem appears unobstructed.

**Figure 3:**
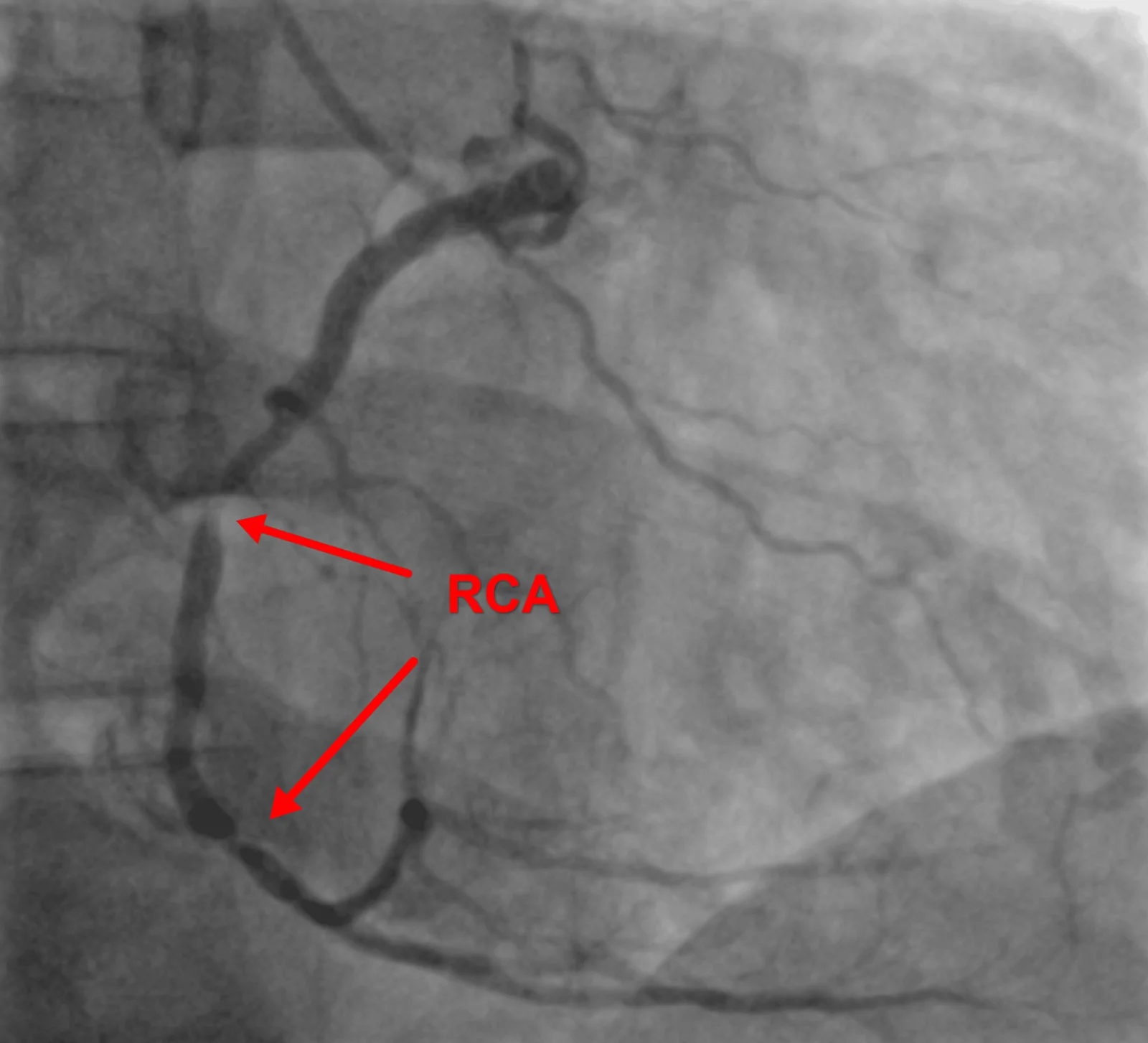
Diagnostic coronary angiography of the dominant right coronary artery (RCA) demonstrating diffuse severe disease involving the mid and distal right coronary artery with severe ostial disease of the posterolateral ventricular branch as pointed out by red arrow.

**Figure 4:**
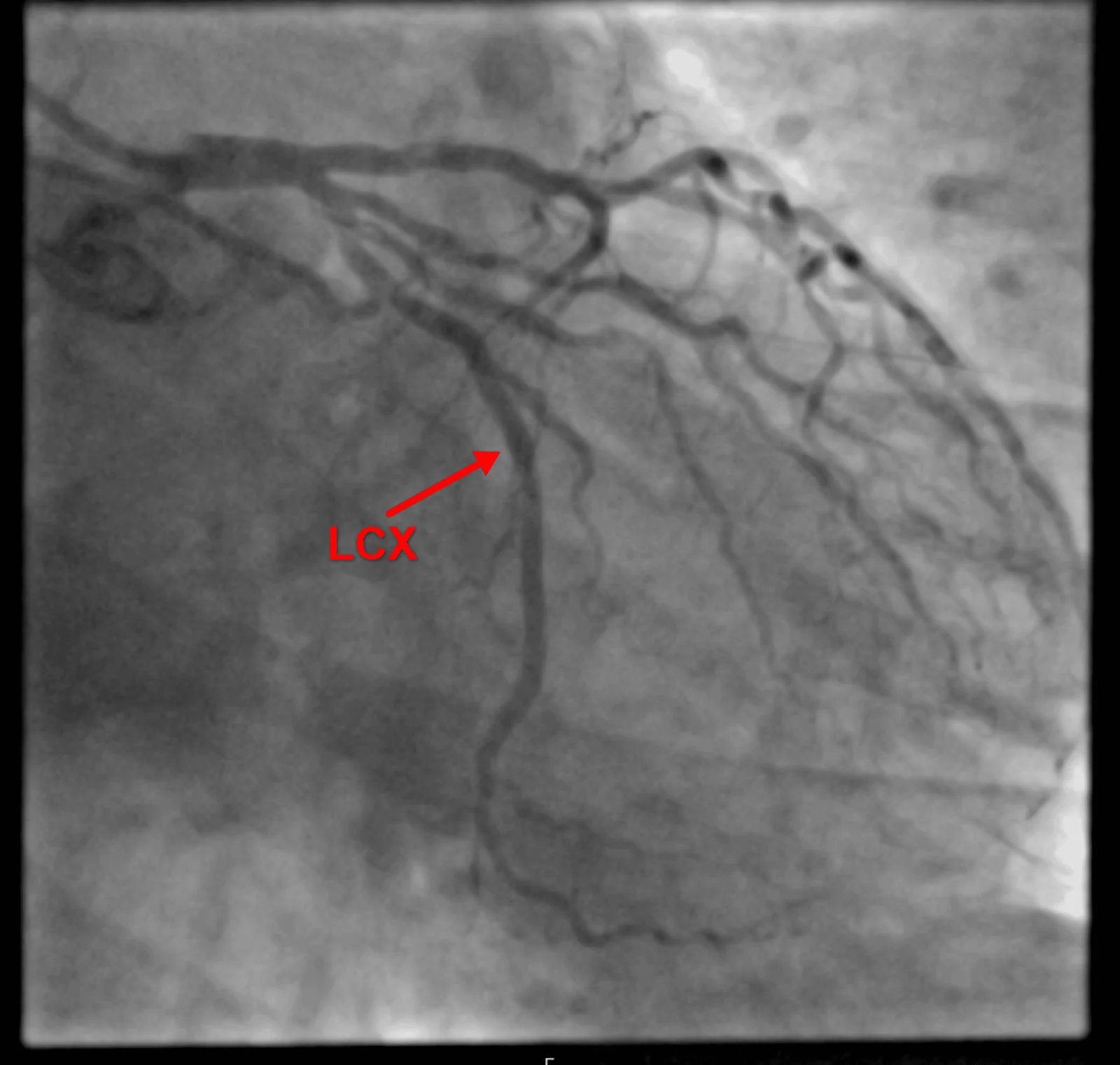
Coronary angiography demonstrating mild disease involving the left circumflex (LCX) coronary artery (red arrow).

Following multidisciplinary discussion, the patient was referred for urgent surgical revascularisation. Ten days after presentation, he underwent coronary artery bypass grafting using the left internal mammary artery to the left anterior descending artery and a saphenous vein graft to the posterior descending artery. His postoperative recovery was uncomplicated. He remained haemodynamically stable throughout his admission, postoperative investigations were satisfactory, and he was discharged home with optimisation of secondary prevention therapy and arrangements for routine cardiothoracic outpatient follow-up. 

## Discussion

Tirzepatide is a first-in-class dual GIP and GLP-1 receptor agonist that has demonstrated remarkable efficacy in the treatment of obesity and type 2 diabetes mellitus [1]. Clinical trials have consistently shown substantial reductions in body weight, improved glycaemic control, and favourable effects on several cardiovascular risk factors, including blood pressure, lipid profile, and systemic inflammation [1,2]. These metabolic improvements are generally expected to translate into lower cardiovascular risk rather than an increased incidence of acute coronary events [2]. 

The present case describes a 48-year-old man who experienced approximately 30 kg of weight loss during 18 months of tirzepatide therapy before presenting with an NSTEMI and being diagnosed with severe triple-vessel coronary artery disease requiring urgent coronary artery bypass grafting. Although the temporal relationship between tirzepatide therapy and the cardiac event is noteworthy, the angiographic findings strongly suggest that advanced multivessel atherosclerotic disease had developed long before presentation. Given the presence of extensive obstructive coronary artery disease, it is more likely that the patient's atherosclerotic burden predated initiation of tirzepatide therapy. 

Several alternative explanations should be considered. First, the patient possessed multiple established cardiovascular risk factors, including South Asian ethnicity, previous obesity, prediabetes, a history of smoking, and a family history of premature coronary artery disease. These factors substantially increase lifetime cardiovascular risk and may have contributed to the development of severe but previously asymptomatic multivessel coronary artery disease [2,5]. 

Second, significant weight loss may have improved the patient's functional capacity, enabling him to undertake higher-intensity physical activity than previously tolerated. His symptoms initially developed during exercise and resolved with rest, a history highly suggestive of exertional myocardial ischaemia. It is therefore plausible that substantial weight loss and increased exercise capacity unmasked previously silent flow-limiting coronary artery disease rather than directly precipitating plaque rupture. This hypothesis is biologically plausible and better explains the clinical chronology than a direct pro-atherogenic effect of tirzepatide, for which there is currently no supporting evidence. 

Current cardiovascular outcome data do not suggest an increased risk of major adverse cardiovascular events with tirzepatide. In the SURPASS-CVOT trial, tirzepatide was noninferior to dulaglutide for the composite outcome of cardiovascular death, myocardial infarction, or stroke in patients with type 2 diabetes and established atherosclerotic cardiovascular disease [3]. Separately, the SELECT trial demonstrated that semaglutide, a GLP-1 receptor agonist, reduced major adverse cardiovascular events in patients with overweight or obesity and established cardiovascular disease but without diabetes [6]. Although these findings support the cardiovascular benefits of GLP-1 receptor agonist therapy in selected populations, they should not be directly extrapolated to tirzepatide. Consequently, the available tirzepatide-specific cardiovascular outcome data do not support attributing this patient's NSTEMI directly to tirzepatide.

Published reports of ACS occurring during treatment with licensed tirzepatide remain extremely limited. A recent conference abstract described ST-elevation myocardial infarction following the use of compounded tirzepatide; however, the formulation differed from licensed tirzepatide preparations, limiting the generalisability of those findings [4]. Continued pharmacovigilance and reporting of similar cases remain important as the use of incretin-based therapies continues to expand worldwide. 

Several limitations should be acknowledged. As a single case report, no causal relationship between tirzepatide therapy and ACS can be inferred. Furthermore, intravascular imaging was not performed to characterise plaque morphology, and the patient's underlying cardiovascular risk profile provides a plausible alternative explanation for his presentation. Consequently, this report should be regarded as hypothesis-generating rather than evidence of a novel adverse drug reaction. 

Nevertheless, this case carries an important clinical message. Patients who achieve substantial weight loss with highly effective anti-obesity therapies should not be presumed to have a low likelihood of obstructive coronary artery disease. The development of new exertional chest pain, dyspnoea, or reduced exercise tolerance should prompt timely cardiovascular assessment irrespective of improvements in body weight or metabolic health. This is particularly relevant in individuals with conventional cardiovascular risk factors or a family history of premature coronary artery disease. Early recognition of these symptoms may facilitate timely diagnosis of clinically significant coronary artery disease before catastrophic cardiac events occur.

## Conclusions

In conclusion, this case demonstrates a temporal association between substantial weight loss during tirzepatide therapy and presentation with NSTEMI secondary to previously undiagnosed severe triple-vessel coronary artery disease. While causality cannot be inferred, this report emphasises the need for continued vigilance when evaluating new cardiovascular symptoms in patients receiving GLP-1/GIP receptor agonists and highlights the importance of further pharmacovigilance studies to determine whether similar observations represent coincidence or a reproducible clinical pattern. 
